# Beyond pneumoperitoneum: technical feasibility and predictive factors for gasless laparoscopic cholecystectomy in dogs

**DOI:** 10.1007/s11259-026-11297-y

**Published:** 2026-06-03

**Authors:** Amanda Oliveira Paraguassú, Otávio Henrique de Melo Schiefler, Raquel Baumhardt, Juan A Sánchez-Margallo, Francisco M Sánchez-Margallo, Vinicius da Silva Cadiñanos, Gabriel Satoru Ohashi, Franciéli Mallman Pozzobon, Daniel Curvello de Mendonça Müller, Maurício Veloso Brun

**Affiliations:** 1 Department of post-graduation, Centro de Ciências Rurais, Universidade Federal de Santa Maria, Santa Maria, RS Brazil; 2 Hospital Veterinário Universitário, Universidade Federal de Santa Maria, Santa Maria, RS Brazil; 3 Jesús Usón Minimally Invasive Surgery Centre (CCMIJU), Cáceres, Spain; 4 Department of small animal clinic, Centro de Ciências Rurais, Universidade Federal de Santa Maria, Santa Maria, RS Brazil

**Keywords:** Gallbladder mucocele, Minimally invasive surgery, Surgical time, Hepatobiliary disease

## Abstract

This study evaluated the safety and feasibility of gasless laparoscopic cholecystectomy in dogs, identifying factors associated with surgical time and survival. Twenty-five dogs with symptomatic gallbladder disease—predominantly gallbladder mucocele (72.0%) and cholelithiasis—underwent cholecystectomy using a mechanical abdominal wall lift system. Variables including gallbladder volume, age, body condition score, and intraoperative complications were recorded. Statistical analyses employed Pearson’s chi-squared, Spearman’s correlation, regression analysis for surgical time predictors, and Kaplan-Meier survival analysis with log-rank tests. No significant association was found between breed and biliary condition. However, surgical time was significantly and positively correlated with body weight and body condition score (*p* < 0.001). Linear regression indicated that each 1 kg increase in body weight added approximately 4 min to the procedure. Gallbladder volume did not correlate with surgical duration. Survival analysis revealed that dogs with gallbladder mucocele had a significantly lower probability of overall survival compared to cholelithiasis (*p* = 0.014). The results demonstrate that the gasless laparoscopic cholecystectomy is a viable and safe alternative for canine patients, especially those at a high cardiorespiratory risk. While increased body weight is a critical predictor of prolonged surgery, the primary biliary pathology remains the most significant determinant of survival.

## Introduction

Gallbladder mucocele (GBM) and cholelithiasis (GC) are the most common canine biliary diseases requiring cholecystectomy (Jaffey 2022). GBM is characterized by the abnormal accumulation of semisolid mucoid bile, often associated with cystic mucosal hyperplasia. This condition can progress to biliary obstruction, mural necrosis, and spontaneous rupture leading to life-threatening bile peritonitis (Pike et al. 2004; Gookin et al. 2025). Laparoscopic cholecystectomy offers a minimally invasive alternative to open surgery, offering reduced postoperative pain., shorter hospitalization (Bleedorn et al. 2013), and lower intraoperative mortality rates – reported at approximately 5.3% compared to 19.6% in open procedures (Kanai et al. 2018). However, conventional laparoscopy relies on carbon dioxide (CO_2_) pneumoperitoneum to create a working space. While generally safe, CO_2_ insufflation can cause significant cardiorespiratory compromise in elderly or systemically compromised patients (Duerr et al. 2008; Mayhew et al. 2013). To address these limitations, the gasless laparoscopic technique utilizing a mechanical abdominal wall lift system was developed to create a workspace without CO_2_ insufflation (Fransson et al. 2015; Milech et al. 2023). Although the feasibility of a new platform for gasless surgery (Brun et al. 2019) has been explored in experimental models (Linhares 2021; Brun et al. 2022; Milech et al. 2023), clinical documentation of its application in complex procedures like cholecystectomy remains limited. This study aims to evaluate the safety and feasibility of this technique using the VET 90,000 platform (BhioSupply^®^) and identify predictors of surgical outcomes.

## Materials and methods

Medical records of 25 dogs (2024–2025) undergoing gasless laparoscopic cholecystectomy in a teaching hospital were retrospectively analyzed. The study included canine patients diagnosed with symptomatic GB disease and data included hematology, abdominal ultrasonography, bacteriological, and histopathological findings, as well as surgical complications (hemorrhage and gallbladder rupture). Patients were categorized into two groups: GBM group (*n* = 18) and GC group (*n* = 7). Ultrasonography grading of GBM (degrees I-V) was performed based on literature (Choi et al. 2014). Recorded variables incleded breed, sex, age, body condition score (BCS) (using a 1–9 scale) (Laflamme 1997). Only feline patients were excluded from this study.

**Fig. 1:**
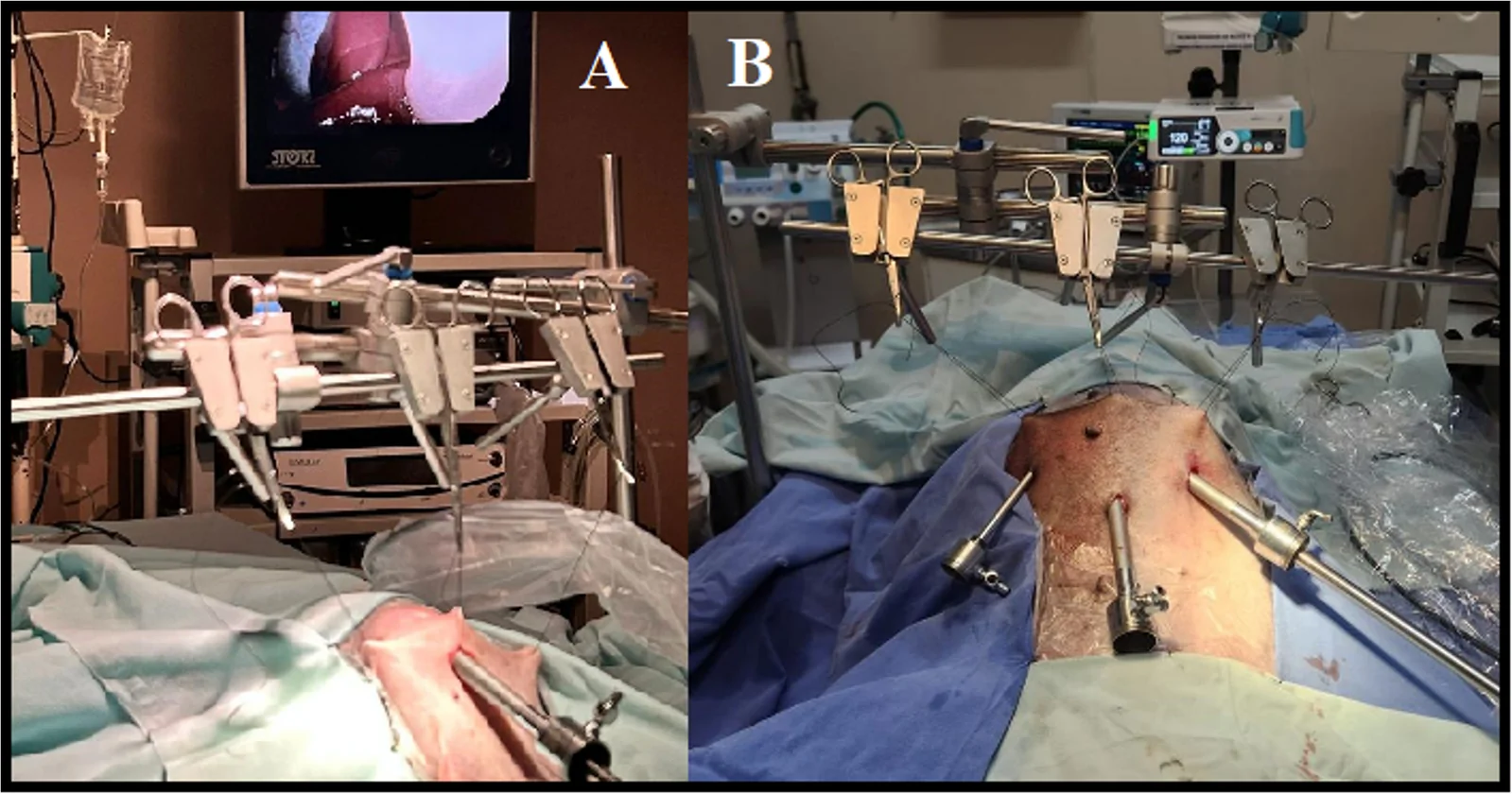
Positioning of the gasless platform (VET 90000) and support sutures for laparoscopic cholecystectomy in a dog. **A** Placement of the first portal with the three transparietal support sutures of the platform. **B** Final aspect of the platform positioning with three-portal assessment

All dogs underwent cholecystectomy using the VET 90,000 platform (BhioSupply^®^) via dorsal recumbency and a three-portal approach with cystic duct clipping and Ligasure^®^ dissection (Brun et al. 2019). The mechanical lift was achieved through transparietal support sutures attached to the platform (Fig. 1).

Follow-up information was gathered through telephone interviews. For statistical analyses, continuous variables were assessed for normality via the Shapiro-Wilk test. Independent Student’s t-test and Mann-Whitney U tests were used for group comparisons. Correlations between BCS, GB volume, and surgical time were quantified using Spearman’s and Pearson’s correlations. Survival analysis was conducted using the Kaplan-Meier method with log-rank tests (*p* < 0.05).

## Results

The study population comprised 18 GBM (72.0%) and 7 GC (28.0%), with a mean age of 11.14 ± 3.73 years, with no significant age difference between groups (*p* = 0.404). Mixed-breed dogs, Yorkshire, and Poodles were the most represented.

Descriptive evaluation revealed morphologic differences between GBM and GC through ultrasonography, with the mean GB was 14.212 ± 13.653 ml at GBM (*n* = 18) and 16.08 ± 26.508 ml at GC (*n* = 7), with no statistical difference between the groups (*p* = 0.875). The GBM had predominantly thin walls (61.1%) while the GC was distributed between normal and thick walls (57.14% and 28.57% respectively). No statistical difference was observed between the groups (*p* = 0.086). No statistical significance was observed between biliary volume and surgical time in the Spearman test (*p* = 0.678), such as the wall thickness (*p* = 0.637). 48.0% of the cases were evaluated in mucocele degree II, followed by degree I (20.0%), degree IV (12.0%), biliary sludge (12.0%) and degree III (8.0%). No statistical correlation was observed with the surgical time (*p* = 0.184).

**Fig. 2:**
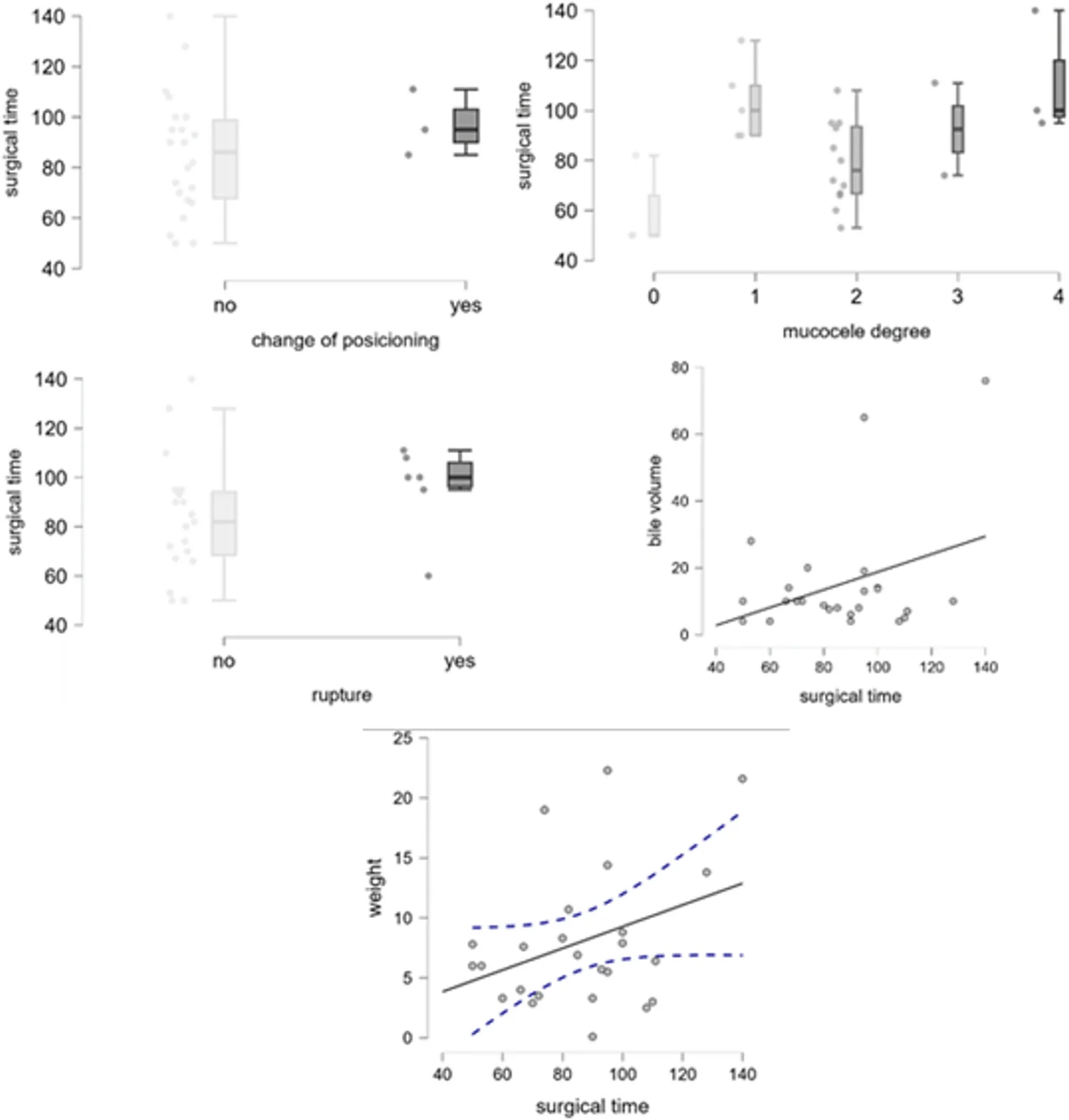
Analysis of surgical time variation across operative and clinical factors in 25 gasless laparoscopic cholecystectomies in dogs. Boxplots and Scatter plots illustrating the distribution of the surgical time in minutes relative to four key procedural and clinical variables. Top Left: Comparison of surgical time based on the necessity of intraoperative change of positioning of the patient. Top right: surgical time distribution stratified by mucocele degree (grades 0 to 4), where the boxes represent the median and interquartile range. Middle left: Mean surgical time (with standard deviation bars) demonstrating the impact of gallbladder rupture (yes vs. no), confirming that rupture significantly prolonged the procedure. Middle right: Scatter plot showing surgical time variation across different categories of bile volume (in mL), revealing no clear linear relationship or significant constraints on surgical time from gallbladder contents. Bottom: The plot displays the relationship between surgical time and body score condition (using a 9-point scale), which also shows a weak positive trend. The solid line represents the regression line, and the dashed lines indicate the 95% confidence interval of the prediction

In the GBM group hepatic hemorrhage was observed in 3 dogs (16.6%), GB rupture in 5 dogs (27.7%), change of positioning during surgery (reverse Trendelenburg 11^o^ to 13^o^) in 3 (16.6%), association with capnoperitoneum in 1 (5.5%), extracorporeal ligature necessity in 2 (11.1&), and association with intestinal biopsy in 2 (11.1%). Only hepatic hemorrhage was observed in the GC group (28.57%). Mean surgical time between GBM and GC was similar (*p* = 0.880): 86.22 min for GBM and 87.43 min for GC. Despite its morphological complexity (high degrees and duct dilation),

The mean surgical time was 86.22 min for the GBM group and 87.43 min for the GC group. A positive significant correlation was identified between BCS and surgical duration (*r* = 0.865; *p* < 0.001) (Fig. 2). A simple linear regression indicated that for each 1 kg increase, surgical time increased by approximately 4 min (*p* < 0.001, β = 4.004). Interestingly, neither GB volume (*p* = 0.678) nor the degree of mucocele (*p* = 0.184) showed a statistical correlation with surgical time. Intraoperative complication such as hepatic hemorrhage (*n* = 5) was not significantly associated with increased surgical duration (*p* = 0.138 and *p* = 0.175, respectively). However, gallbladder rupture tended to prolong operative time due to the need for abdominal lavage and additional intraoperative management (Fig. 2).

Bacterial growth was observed in 16.0% of the dogs, 75.0% of which were multi-resistant. One *Pseudomonas aeruginosa*-resistant growth and one *Enterococcus* sp. growth were observed in the GBM group (11.11%), and both patients had rupture of the gallbladder as a complication. One *Acinetobacter* spp. resistant growth and one *Klebsiella* spp. resistant growth were observed in GC (28.57%), with both not presenting gallbladder rupture.

**Fig. 3:**
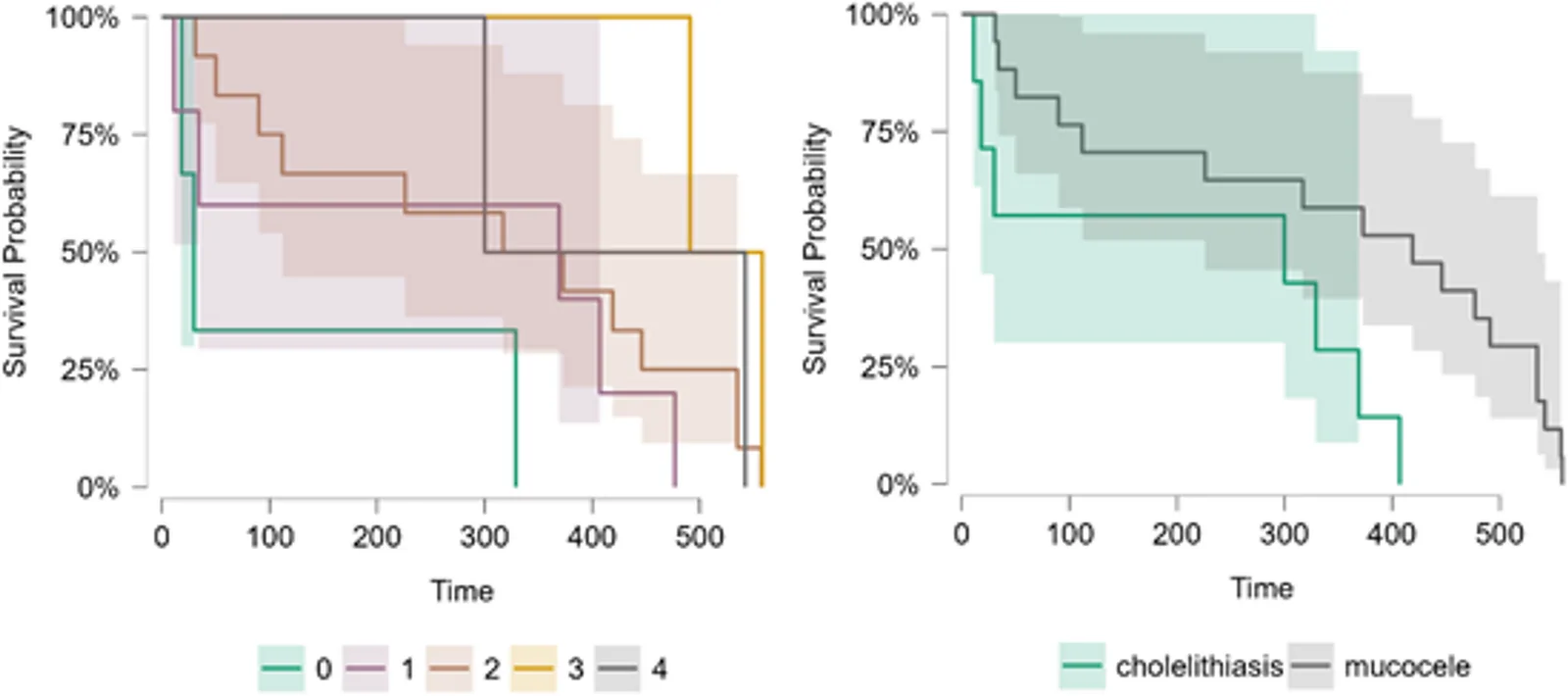
Kaplan-Meier survival analysis following laparoscopic cholecystectomy in dogs, stratified by clinical and surgical factors. Kaplan-Meier survival plots illustrating the probability of overall survival (y-axis) over time (x-axis) following gasless laparoscopic cholecystectomy in dogs. Top left: survival probability stratified by mucocele degree (grades 0 to 4) (p=0.134). Top right: survival probability stratified by the primary biliary condition: cholelithiasis vs. mucocele. The two curves demonstrate a statistically significant difference in overall outcomes between the two primary diseases in the postoperative period (p=0.037). Patients with mucocele showed a lower survival probability in the postoperative follow-up period

Kaplan-Meier analysis revealed a significant difference in survival based on primary biliary pathology (Fig. 3), where GBM exhibited a significantly lower probability of overall survival compared to the GC group (log rank test: *p* = 0.014). A single non-surgical death occurred 14 days post-procedure due to unrelated systemic disease (leishmaniasis).

## Discussion

This study addresses the clinical application of gasless laparoscopic cholecystectomy for treating canine GB disease. The primary advantage of this approach is the mitigation of cardiorespiratory risks associated with CO_2_ pneumoperitoneum, which is crucial for the geriatric population often affected by GBM and GC (Parkanzky et al. 2011). Additionally, the gasless approach facilitates the drainage of abdominal lavage fluid without looing the visual field and allows the use of valveless access ports, which enhances instrument maneuverability. The absence of statistically significant association between GB volume and surgical time (*p* = 0.678) suggests a high degree of adaptability of the gasless approach across varying gallbladder conditions. The surgical field, independently of the organ volume, allowed a safe dissection, corroborating with previous studies for advanced procedures (Linhares 2021).

However, the interpretation of these advantages should be made with caution. In the absence of a control group undergoing capnoperitoneum, it is not possible to directly compare the physiological and surgical benefits of the gasless technique. Therefore, any assumptions regarding its superiority remain speculative and should be validated in prospective controlled studies.

Furthermore, bacterial growth was identified in 16.00% of the dogs, with 75% being multi-resistant, is consistent with reports indicating high rates of ascending bacterial infection in the biliary system (Galley et al. 2022). In this report, two of these cases presented GB rupture, which needed abdominal lavage but didn’t lead to biliary peritonitis.

The most striking finding was the strong correlation between BCS and surgical time (*r* = 0.865; *p* < 0.001). In overweigh or larger dogs, the increased thickness of the abdominal wall and the presence of abundant intra-abdominal fat create technical challenges such as instrument maneuvering and visualization. Surgeons should anticipate longer anesthesia times when performing this procedure in obese patients.

Interestingly, GB volume and mucocele degree did not influence surgical time. This suggests that the gasless platform provides consistent workspace regardless of the size of the organ being removed. Even in cases of degree IV BM, where the content is highly organized and the wall may be compromised, the gasless approach allowed for safe dissection.

Although the difference in surgical time was not statistically significant (*p* = 0.120), intraoperative complications, particularly GB rupture, led to longer procedures due to the need for extensive peritoneal lavage. However, the gasless technique offered a unique benefit: the ability to perform abdominal lavage without the loss of the visual field. Despite the higher frequency of multi-resistant bacteria (75%) as described by Galley et al. (2022), the use of intense lavage prevented the development of postoperative biliary peritonitis.

The significant lower survival rate (*p* = 0.014) in the GBM group compared to the GC group suggests that long-term prognosis was primarily driven by the underlying disease rather than the surgical technique. This is likely attributable to the contribution of systemic metabolic or endocrine dysfunctions, which may lead to systemic failure even after resolution of the mechanical obstruction (Galley et al. 2022). Nevertheless, the imbalance in sample size between disease groups may limit the generalizability of these findings, and different outcomes could emerge in larger cohorts of cholelithiasis cases.

The retrospective nature and the relatively small sample size, particularly in the GC group, limit the statistical power for some comparisons, Furthermore, the lack of a control group undergoing capnoperitoneum prevents a direct physiological comparison of the two methods.

Gasless laparoscopic cholecystectomy is a secure minimally invasive option for managing canine biliary disease. While increased BCS significantly prolong surgical time, they do not prelude the use of the technique. The primary determinant of patient survival is the nature of the biliary disease itself, with GBM patients requiring more intensive long-term management.

## Data Availability

No datasets were generated or analysed during the current study.
